# Circulating CD81-expressing extracellular vesicles as biomarkers of response for immune-checkpoint inhibitors in advanced NSCLC

**DOI:** 10.3389/fimmu.2022.987639

**Published:** 2022-09-20

**Authors:** Diego Signorelli, Patrizia Ghidotti, Claudia Proto, Marta Brambilla, Alessandro De Toma, Roberto Ferrara, Giulia Galli, Monica Ganzinelli, Giuseppe Lo Russo, Arsela Prelaj, Mario Occhipinti, Giuseppe Viscardi, Valentina Capizzuto, Francesca Pontis, Ilaria Petraroia, Anna Maria Ferretti, Mario Paolo Colombo, Valter Torri, Gabriella Sozzi, Marina Chiara Garassino, Elena Jachetti, Orazio Fortunato

**Affiliations:** ^1^ Thoracic Oncology Unit, Fondazione IRCCS Istituto Nazionale dei Tumori, Milan, Italy; ^2^ Tumor Genomics Unit, Fondazione IRCCS Istituto Nazionale dei Tumori, Milan, Italy; ^3^ Sezione Via G. Fantoli 16/15, Istituto di Scienze e Tecnologie Chimiche-CNR, Milan, Italy; ^4^ Molecular Immunology Unit, Fondazione IRCCS Istituto Nazionale dei Tumori, Milan, Italy; ^5^ Oncology Department, Istituto ‘Mario Negri’ – IRCCS, Milan, Italy

**Keywords:** lung cancer, extracellular vesicles (EV), PD-L1, CD81 (tetraspanin), immunotherapy

## Abstract

PD-L1 in tumor cells is the only used biomarker for anti PD1/PD-L1 immune-checkpoints inhibitors (ICI) in Non Small Cell Lung Cancer (NSCLC) patients. However, this parameter is inaccurate to predict response, especially in patients with low tumor PD-L1. Here, we evaluated circulating EVs as possible biomarkers for ICI in advanced NSCLC patients with low tumoral PD-L1. EVs were isolated from plasma of 64 PD-L1 low, ICI-treated NSCLC patients, classified either as responders (R; complete or partial response by RECIST 1.1) or non-responders (NR). EVs were characterized following MISEV guidelines and by flow cytometry. T cells from healthy donors were triggered *in vitro* using patients’ EVs. Unsupervised statistical approach was applied to correlate EVs’ and patients’ features to clinical response. R-EVs showed higher levels of tetraspanins (CD9, CD81, CD63) than NR-EVs, significantly associated to better overall response rate (ORR). In multivariable analysis CD81-EVs correlated with ORR. Unsupervised analysis revealed a cluster of variables on EVs, including tetraspanins, significantly associated with ORR and improved survival. R-EVs expressed more costimulatory molecules than NR-EVs although both increased T cell proliferation and partially, activation. Tetraspanins levels on EVs could represent promising biomarkers for ICI response in NSCLC.

## Introduction

Improvements for lung cancer prevention, diagnosis and treatment have been observed in the past 20 years, however lung cancer still remains the leading cause of cancer-related deaths (1). Nowadays, the estimated 5-year survival rate for non-small-cell lung cancer (NSCLC) patients is around 20%. Surgery represents the standard of care for early-stage NSCLC and the only treatment with curative intent; however only 60% of stage IB, 50% of stage II and 40% of stage IIIA subjects survive at 5 years (1).

Immunotherapy has radically changed the treatment paradigm in NSCLC. Since 2015, immune checkpoint inhibitors (ICI) blocking PD1/PD-L1 axis have been approved in pretreated metastatic NSCLC (2, 3). Data from phase III trials have showed ICI efficacy in early and locally advanced stages, with durvalumab approved as maintenance treatment after definitive chemoradiotherapy (4) and nivolumab and atezolizumab showing activity in the neoadjuvant (5) and adjuvant (6) settings, respectively. Despite the clinical application, a gap of knowledge still exists regarding predictive biomarkers. So far, Programmed Death Ligand 1 (PD-L1) expression on tumor cells is the only pathologic biomarker approved in clinical practice. Tumor mutational burden (TMB) has recently emerged as a new promising biomarker (7). Higher TMB showed a positive predictive value for ICI alone in retrospective (8) and prospective studies (9, 10); however, lower concordance was observed between TMB and PD-L1 expression (11). Furthermore, a clear cut-off still misses, no predictive role for TMB has emerged in prospective trials with ICI plus chemotherapy (12), and the high costs of TMB analysis definitively increase the risk of financial toxicities. For these reasons, easily measurable and reproducible biomarkers based on clinical and biochemical features (13–16) have been looked for. Although PD-L1 expression is not a perfect predictive factor, around 32% of NSCLC patients with high PD-L1 expression who received first line pembrolizumab survive at 5 years (17). However, patients with low PD-L1 expression have shown to be potential responders to ICI (3) and the response rate of patients with negative PD-L1 values on tumor cells is around 15% (18). Therefore, the lack of biomarkers negatively affects mainly patients with negative and low PD-L1 expression. Exploratory studies tried to identify biomarkers of response by analyzing the cells composing the immune infiltrate of the tumors (19). Indeed, the presence of tumor infiltrating CD8^+^ lymphocytes was correlated with PFS and response rate in NSCLC patients treated with anti-PD1 (20). However, the limitation of the use of these promising cellular markers is the lack of surgical tissues for advanced NSCLC patients. The analysis of circulating immune cells or soluble factors in the blood now represents an interesting field for the discovery of new biomarkers of response to therapy. Indeed, neutrophils to lymphocytes ratio or the absolute count of circulating neutrophils were negative prognostic factors in lung cancer patients treated with ICI (21, 22). Larger studies are needed to better define the impact of these new identified circulating biomarker in clinical practice.

Extracellular vesicles (EVs) are an evolutionarily conserved group of bilayered membrane vesicles and are classified by size and intracellular origin into two main categories: small EVs (sEVs, 50–150 nm) and microvesicles (MVs 100 nm–1 mm) (23). EV’s were observed to induce modulation of vascular permeability and neo-angiogenesis allowing and supporting cell extravasation and metastatic outgrowth (24). Furthermore, the functional cargo of EVs comprises proteins, lipids and nucleic acids such as DNA and RNA (mRNAs, lncRNAs, and miRNAs) (25), all able to modulate several biological processes in recipient cells (26). Due to their intrinsic characteristics EVs could be considered a reliable and stable biomarker in the circulation and, in particular, their surface proteins could be useful for the diagnosis of the disease. Indeed, PD-L1 on the surface of EVs circulating in the blood of patients with head and neck cancer and melanoma has been associated with tumor progression (27, 28). In addition, the level of PD-L1 on exosomes collected at baseline and during therapy correlates with ICI responses and survival in melanoma patients treated with anti-PD1 (27). These exosomes were shown to be as immunosuppressive as cancer cells are for the inhibition of T-cell activation (29). In lung cancer, the immunosuppressive activity of EV-PD-L1 against T cells was tested only *in vitro* using exosomes isolated from commercial lung cancer cell lines (30). However, in NSCLC the role of PD-L1 and other surface markers on EVs both as biomarker of patients’ outcome and in mediating immunosuppression is largely unknown. In this study, we characterized the biological and functional features of circulating EVs isolated from NSCLC patients treated with ICI, to find new potential biomarkers of response.

## Material and methods

### Cell lines

The human LT73 lung cancer cell line was derived in our laboratory from a primary lung tumour of a 68-year-old male and was cultured in RPMI 1640 medium (Gibco, Thermo Fisher Scientific, Waltham, MA, USA) supplemented with 10% foetal bovine serum (FBS; EuroClone, Italy) and 1% penicillin-streptomycin (Sigma-Aldrich, Saint Louis, MO, USA).

### Clinical characteristics of NSCLC patients

Plasma samples were collected from 64 pts: 26 (40.6%) with Tumor Proportion Score (indicating the percentage of tumor cells stained positive for PD-L1 in tissue, TPS) low (1-49%) and 38 (59.4%) with absent TPS (<1%); they were treated with ICI as first (n=26) or further (n=38) line. Patients were classified in responders (R, 21.7%) if they achieved a complete or partial response by RECIST 1.1, non-responders (NR, 78.3%) otherwise (**Supplementary Tables 1–3** and **Supplementary Figure 1**). For the control group plasma were collected from high-risk heavy-smoker volunteers (HS, age 50–75 years), including current or former smokers with a minimum pack/year index of 30 enrolled in a LDCT screening trial (BioMild Trial, ClinicalTrials.gov: NCT02247453) (31). The study was conducted according to the criteria set by the declaration of Helsinki and all patients provided informed consent. Plasma collection was approved by the Internal Review and the Ethics Boards of the Istituto Nazionale dei Tumori (INT 22-15 and INT 11-21) of Milan.

### EVs isolation

Plasma EVs were obtained from whole blood and purified by differential centrifugation processes as previously described (32, 33). In brief, EVs were isolated from 1 **ml** of stored plasma by ultracentrifugation at 120000 × g and 4°C for 90 minutes using a TLA-100.3 fixed-angle rotor in a TL-100 ultracentrifuge (Beckman Coulter, Brea, CA, USA). EV-enriched pellet was washed in phosphate-buffered saline (PBS; Thermo Fisher Scientific) at 120000 × g for 60 minutes at 4°C to remove impurities and then resuspended in PBS. The protein content of the purified EVs was determined by the Bradford assay.

LT73-derived EVs were isolated starting from their conditioned medium (CM). At confluence, complete medium of cells was removed and replaced with fetal bovine serum-free medium. After 48h, CM of cells was collected and centrifuged at 3200 x g for 25 minutes in order to remove cell debris. CM was then used for EVs isolation following the same protocol described for plasma EVs.

### EVs characterization

The EV concentration and size distribution were determined by using a NanoSight NS300 instrument (Malvern Panalytical) as described in (33). The videos were analyzed using NTA 3.2 software. EV morphology was assessed using a Zeiss LIBRA 200FE transmission electron microscope with an in-column second-generation Omega filter (33). The size of the EVs**’** was measured by analyzing one hundred EVs using the iTEM imaging platform.

### Flow cytometry on EVs

Flow cytometry analysis was performed with 30 μg of EVs in accordance with the procedures previously described by Théry et al. (34).

Plasma-derived EVs were first incubated with 10 μl of latex beads for 15 minutes at room temperature (RT) and then incubated overnight at 4°C in PBS. After incubation, 100 mM glycine was added to each sample for 30 minutes at RT, and the samples were then stained with the antibodies reported in **Supplementary Table 4** for 15 minutes at RT. Samples were acquired with a BD FACSCanto II instrument (BD Biosciences, San Jose, CA, USA) and analyzed with the FlowJo software (BD Biosciences).

### Flow cytometry on human T cells

T cells were stimulated 4h with PMA (120 ng/ml) and ionomicin (1μg/ml) adding brefeldin A (10μg/ml) in the last 3h. Then, for surface staining, cells were labeled for 15 minutes at 4°C with the desired fluorochrome-conjugated monoclonal antibodies. For intracellular staining, cells were stained for surface markers, fixed (eBioscience™ Intracellular Fixation Buffer, Thermofisher, cat. no. 00-8222-49) and permeabilized (eBioscience™ Permeabilization Buffer, Thermofisher, cat. no. 00-8333-56) before incubation with the desired antibody (**Supplementary Table 4**). Samples were acquired with a BD Celesta instrument (BD Biosciences, San Jose, CA, USA) and analyzed with the FlowJo software (BD Biosciences).

### MACSPlex analysis

Surface marker profiling of plasma-EVs was performed using a MACSPlex Exosome Kit (cat. n. 130-108-813) following manufacturer**’**s instructions (Miltenyi Biotec, Bergisch-Gladbach, Germany). Briefly, 15 μl of MACSPlex Exosome Capture Beads and 15 μl of MACSPlex Exosome Detection Reagent cocktail (CD9, CD63, and CD81) were added to each sample (15 μg of EVs). Samples were incubated at 4°C in the dark for 1 **h**, washed two times with 500 μl of MACSPlex Buffer and analyzed by flow cytometry. Acquisition was on a BD FACSCanto II instrument (BD Biosciences, San Jose, CA, USA) and analysis was performed with FlowJo software (BD Biosciences).

### T cell proliferation assays

Peripheral blood mononuclear cells (PBMCs) were obtained from whole blood samples of healthy subjects through a density gradient stratification. Briefly, blood was collected in heparin, then diluted 1:2 in PBS and layered onto Histopaque-1077 Ficoll (Sigma- Aldrich). The blood:ficoll ratio was 1:1. Samples were centrifuged at 1800 rpm for 30min at room temperature without brake. The lymphocyte-enriched ring formed at the interface was transferred into a new collection tube and washed two times with PBS.

Total T cells (CD4+ and CD8+) were purified from PBMCs with the Pan T Cell Isolation Kit (Miltenyi Biotec, cat. no. 130-096-535) according to the manufacturer’s protocol and seeded overnight with 20 IU/ml of recombinant human IL2 (Miltenyi Biotec, cat. no. 130-097-746). The following day T cells were labeled with 2.5 μM CFSE (Thermofisher, cat. no. 65-0850-84) and activated *in vitro* with CD3/CD28 (T Cell TransAct, Miltenyi Biotec, cat. no. 30-111-160), plating 10 (5) cells in each well of a 96 well plate. When indicated, cell-or patient-derived EVs were added (15 µg/well). After 7 days, cells were analyzed for proliferation (measured as CFSE dilution) and activation by flow cytometry.

### Statistical analysis

Descriptive statistics of patients’ demographics (eg, age, sex) and clinical characteristics (eg, stage, therapy) were reported as frequencies (proportions) for categorical variables and mean and STD or median (IQR) for continuous variables. Summary measures for association between demographic and clinical characteristics, and outcomes were assessed by univariable logistic models. Results are given as odds ratios (ORs) with 95% CIs. Regarding EV and other biomarker characteristics, given an expected high grade of correlation a reduction strategy was implemented before running multivariate analysis. A clustering procedure was used and a further multivariable logistic regression for the association between clusters and different outcomes was performed. The variables of cluster associated with outcome were analyzed in multivariate analysis (logistic for response outcome Cox Model from time to events outcomes) with a fast-backward step-down selection. Since the study analysis was based on a convenience sample, no power analysis was done to calculate the sample size.

For *in vitro* experiments, the statistical analysis was performed using Graphpad Prism 5 software. Statistically significant differences were determined with Student’s t‐test when comparing two groups or ANOVA test for multiple comparisons

## Results

### Characterization of EVs from NSCLC patients treated with anti-PD1 therapy

**Figure 1:**
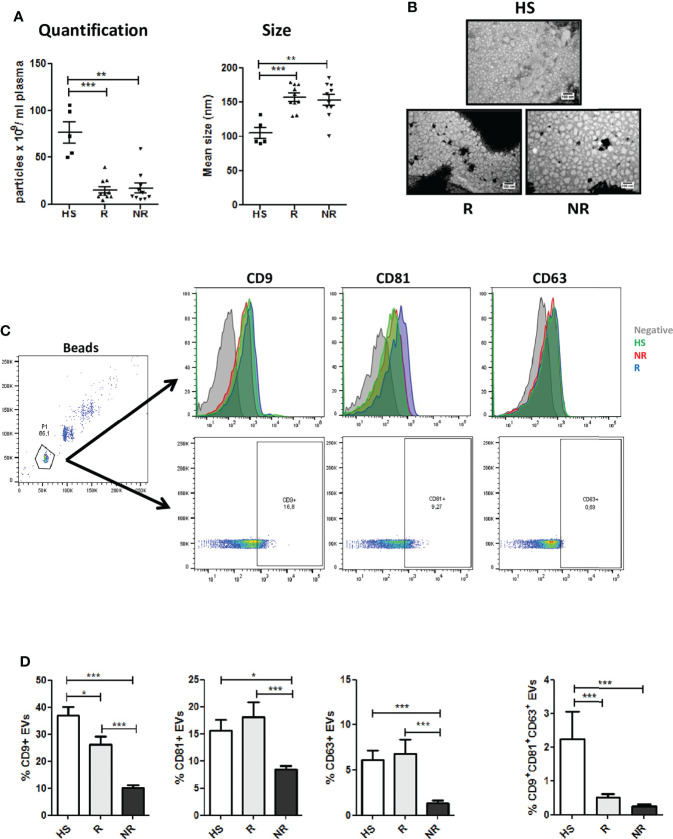
Characterization of plasma EVs from NSCLC patients at baseline. **(A)** Concentration and size distribution of EVs from Responder (R) compared to EVs from Non Responder (NR) patients and heavy smokers (HS) healthy controls, using nanoparticle tracking analysis (n = 10 per group). **(B)** Representative TEM images showing the spherical morphology and size distribution of plasma-derived EVs. **(C)** Flow cytometric analysis of conventional EV markers (CD63, CD81, CD9) on HS, R and NR-EVs. Representative histogram and dot plots are reported**. (D)** Histogram show the percentages of CD63, CD81, and CD9 positive EVs, or the percentage of CD9/CD81/CD63 triple positive EVs in the three cohorts (HS n = 5; R n = 13; NR n = 48). Data are expressed as mean value ± S.E.M. values. (*p < 0.05; **0.05 < p < 0.001; ***0.001 < p < 0.0001).

Plasma-EVs were characterized in accordance with the Minimal Information for Studies of Extracellular Vesicles (MISEV) guidelines (35). NSCLC patients showed lower amount of total particles if compared to control HS donors. Among patients similar numbers were observed for R and NR samples (**Figure 1A**). Interestingly, nanoparticle tracking analysis revealed a bigger size for EVs isolated from NSCLC patients compared to HS, but again no significant differences in the size distributions of the particles were observed within the two cancer groups (**Figure 1A**). Transmission Electron Microscopy (TEM) analysis revealed that plasma EVs had spherical shapes and a relatively wide size distribution (mean diameter: NR 56.1 ± 15.4 nm vs R 48 ± 7.7 nm) (**Figure 1B**). Furthermore, we assessed the expression of conventional surface EV markers such as CD9, CD81 and CD63 on R and NR EVs *via* flow cytometry. Interestingly, R-EVs expressed significant higher levels of tetraspanins compared to NR-EVs (measured both as number of positive EVs and as Mean Fluorescence Intensity) possibly reflecting differences in the origin of circulating EV populations among the two groups (**Figures 1C, D**, **Supplementary Figure 2A** and **Supplementary Table 5**). The tetraspanin profiles of HS-EVs were similar to EVs isolated from R patients except for CD81 levels (**Supplementary Figure 2A** **)**.

**Figure 2:**
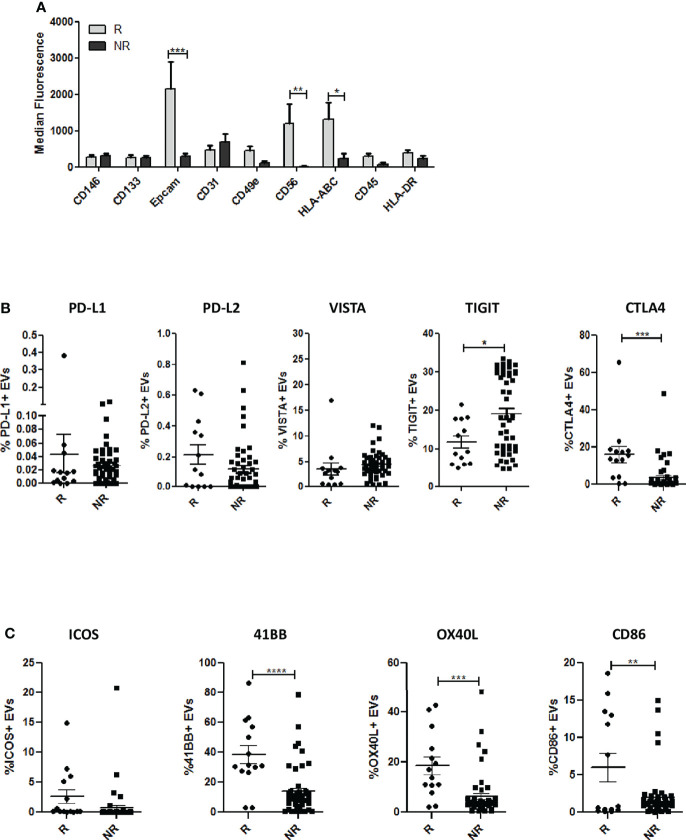
Phenotypic analysis of EVs. **(A)** Profiles of surface markers determined by MACSPlex Exosome Kit in EVs from R and NR patients. The values are the median fluorescence intensities (R n = 8; NR n = 10). **(B)** Surface expression of inhibitory molecules PD-L1, PD-L2, VISTA,TIGIT and CTLA4 on EVs from R (n = 13) and NR patients (n = 51), evaluated by flow cytometry. **(C)** Surface expression of co-stimulatory molecules (CD86, 41BB, ICOS and OX40L) in EVs from R (n = 13) and NR patients (n = 51), measured by flow cytometry. The data are expressed as the mean ± S.E.M. values. (*p < 0.05; **0.05 < p < 0.001; ***0.001 < p < 0.0001; ****p < 0.0001).

To investigate the cell-type specific origin of EVs, we performed multiplex phenotypic analysis using the MACSPlex platform that allows the simultaneous analysis of 37 different surface markers. Differences in tetraspanins detected using conventional flow cytometry (**Figures 1C, D**) between the two groups were also confirmed using this kit (**Supplementary Figure 2B**). Furthermore, this analysis revealed higher levels of EpCAM in R-EVs compared to NR-EVs before the starting of anti-PD-1 treatment (**Figure 2A** and **Supplementary Table 5**). Up-regulation of HLA-ABC and CD56 in R-EVs was also observed (**Figure 2A** and **Supplementary Figure 2C**) whereas no significant changes in the other markers tested were evaluated. We also profiled baseline EVs for the surface expression of co-inhibitory (PD-L1, PD-L2, VISTA, TIGIT and CTLA4) and co-stimulatory (ICOS, 41BB, OX40L and CD86) molecules able to influence activation, proliferation and effector functions of T cells (**Figures 2B, C** and **Supplementary Table 5**). R-EVs were enriched in CTLA4, 41BB, OX40L and CD86 whilst NR-EVs were enriched in TIGIT. We also correlated the expression of PD-L1 in lung cancer tissues with the levels of PD-L1 in circulating EVs. Considering the median values of PD-L1 on EVs no correlation was observed between tissues with TPS <1% or ranging from 1 to 49% or with TPS> 50% (**Supplementary Figure 2D**). Taken together these results suggested that plasma-derived EVs rather than being tumor-derived seemed to arise from the tumor microenvironment reflecting its characteristics. Indeed, high levels of co-stimulatory molecules on the surface of R-EVs could suggest a “hot” tumor milieu, probably more prone to response to ICI therapy.

### Anti-PD1 treatments modulate EV’s phenotype

**Figure 3:**
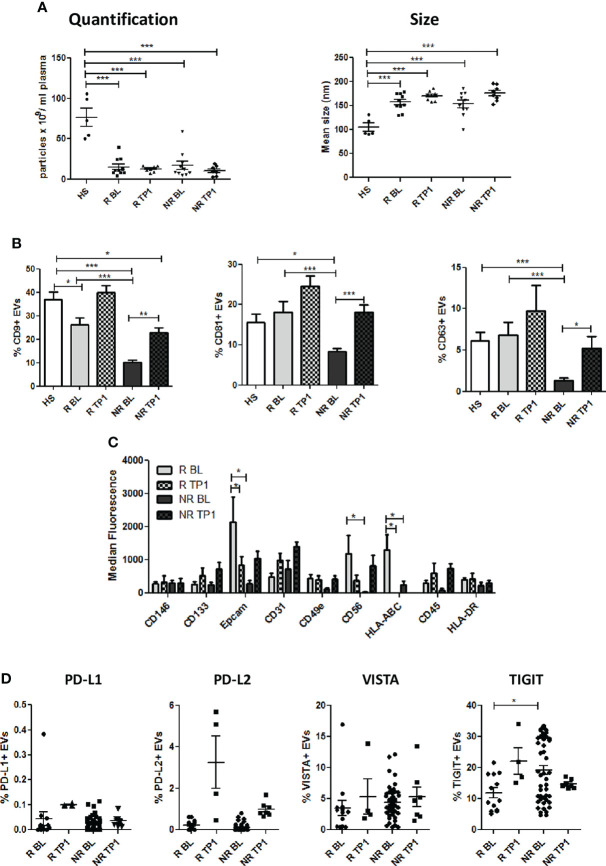
Characterization of plasma EVs from NSCLC patients during therapy. **(A)** Concentration and size distribution of EVs from R and NR patients, collected during ICI therapy, and heavy smokers (HS) healthy controls, using NTA (n = 8 per group). **(B)** Flow cytometric analysis of CD63, CD81, and CD9 EV markers during therapy on R and NR-EVs (R BL n = 13; R TP1 n = 4; NR BL n = 51; NR TP1 n = 7) **(C)** Surface EVs’ markers profiles determined by MACSPlex Exosome Kit in R and NR-EVs patients. The values are the median fluorescence intensities (R BL n = 8; R TP1 n = 6; NR BL n = 10; NR TP1 n=6). **(D)** Surface levels of of PD-L1, TIGIT, PD-L2 and VISTA in EVs collected pre- and after 1^st^ treatment from R and NR patients (R BL n = 13; R TP1 n = 4; NR BL n = 51; NR TP1 n = 7). The data are expressed as the mean ± S.E.M. (*p < 0.05; **p < 0.01; ***0.001 < p < 0.0001).

Next, we examined circulating EVs isolated from patients at baseline (BL) and during anti-PD1 treatment (TP1, range: 8-13 weeks) analyzing size, morphology and phenotype. As shown in **Figure 3A**, EVs had similar total particles count between NR and R and no changes in size were observed (mean size: R 170.8 ± 3.7 nm vs NR 176 ± 5.7 nm). Differences in number and size observed between NSCLC and HS were unaltered during therapy (**Figure 3A** **).** Upon therapy CD9, CD81 and CD63 expression increased in both R and NR EVs (**Figure 3B** and **Supplementary Figure 3A**), hinting a possible influence of therapy on EVs origin. However, despite the increase observed during therapy, levels of these makers still remained lower in NR EVs compared to HS EVs (**Figure 3B**). Profiling 37 specific cell-type surface markers with MACSPlex platform, the differences in EpCAM, CD56 and HLA-ABC expression observed between R and NR in pre-treatment samples, were lost in plasma EVs isolated during therapy (**Figure 3C** and **Supplementary Figure 3B**). Interestingly, anti-PD-1 therapy increased the levels of EVs released by CD14, CD1c and CD25 positive cells (**Supplementary Figure 3B**). Furthermore, during therapy we observed a trend of increase of the levels of PD-L1, TIGIT and PD-L2, albeit not statistically significant, in R-EVs compared to NR-EVs (**Figure 3D**). The variation in tetraspanins expression and the increase of CD14, CD1c and CD25 after therapy suggested a potential influence of ICI on the generation and origin of circulating EVs.

### NSCLC patient-derived EVs do not affect T cell proliferation and activation status

It is known from literature that tumor-derived EVs can affect T cell activation and this correlate with resistance to ICI (27, 29). Thus, we asked if EVs obtained from NSCLC patients could affect T cell activation and effector function. To set up proper *in vitro* conditions, we first set up the experiments utilizing EVs isolated from the LT73 lung cancer cell line. Therefore, we collected CD8^+^ and CD4^+^ T cells from PBMC of healthy donors and activated them with CD3/CD28 specific beads, in presence or not of different concentrations of LT73-derived EVs (1μg, 5 μg, 15μg). We observed that 15μg of LT73-EVs remarkably suppressed proliferation (evaluated as CFSE elution) and GranzymeB production of both CD4+ and CD8+ T cells, whereas production of IFNγ was not affected(**Supplementary Figure 4** **).**

**Figure 4:**
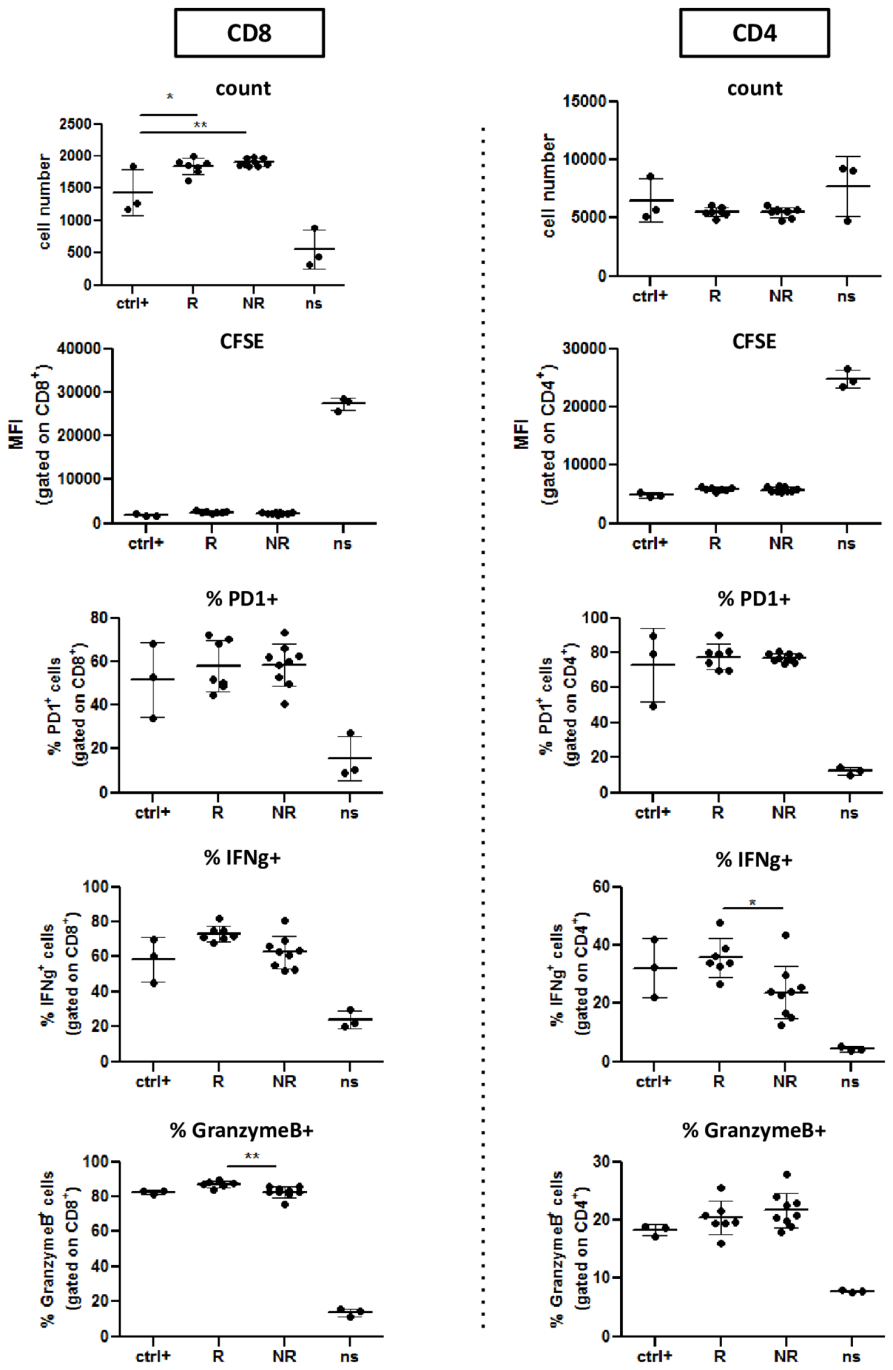
R-EVs and NR-EVs do not affect T cell proliferation and activation. CFSE labeled CD8^+^ and CD4^+^T cells isolated from healthy donor PBMCs were primed *in vitro* with CD3/CD28, either alone (Ctrl+) or in presence of EV from Responder (R) or non responder patients (NR). Negative control T cells were left not stimulated (ns). Histograms show absolute numbers of CD8+ and CD4+ lymphocytes and proliferation (measured as CFSE dilution) and percentage of IFNγ^+^, PD1^+^, and Granzyme B^+^ cells within either CD8+ or CD4+ T cells. *p<0.05; **p<0.01; ns, not stimulated.

Based on these results we decided to use 15μg of EVs as optimal concentration for the next experiments with plasma-EVs. We therefore repeated the described experiments with patient EVs collected at baseline (**Figure 4** and **Supplementary Figure 5**). Strikingly, we did not observe any inhibition of T cell proliferation (CFSE dilution). On the contrary, we observed a slight increase of CD8^+^ T cell numbers in presence of R-EVs (**Figure 4**). Moreover, IFNγ production by CD8+ T cells seemed even boosted by the presence in the culture of EVs, regardless of their origin, while IFNγ production in CD4+ T cells was not affected. Instead, we did not find any difference in PD-1 expression or Granzyme B production (**Figure 4** and **Supplementary Figure 6**).

### CD81 levels on circulating EVs are associated to better response

**Figure 5:**
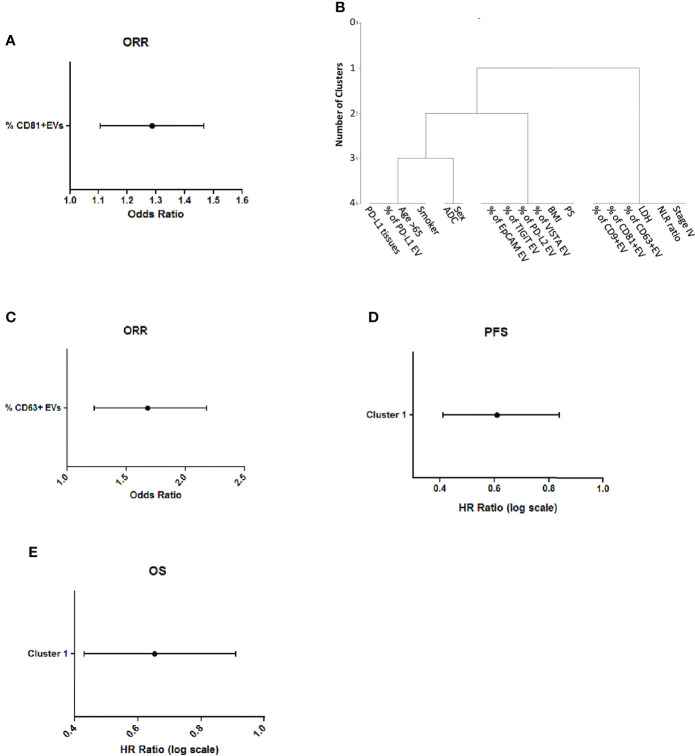
CD81 levels on circulating EVs are associated to better response and survival **(A)** Multivariable logistic analysis of association between variables selected from univariable analysis **(B)** Representation of cluster procedure selection variables in our cohort of patients **(C)** Association of CD63 + EVs with ORR **(D)** Association of cluster 1 with Progression Free Survival in lung cancer patients **(E)** Association of cluster 1 with Overall Survival of lung cancer patients.

In order to identify a prognostic biomarker of ICI response, with an unsupervised approach we analyzed all the variables associated to patients or EVs features (**Supplementary Table 5**) in relation to Overall Response Rate (ORR) and we found that the percentage of CD9, CD63 and CD81-EVs were significant associated to a better ORR (**Supplementary Table 6**). Among these variables the number of CD81 positive EVs was significant in multivariable logistic analysis (**Figure 5A**).

Then, we also applied a clustering procedure to all the described variables, to find which of them were closely correlated with each other. We obtained three different clusters (**Figure 5B** and **Supplementary Table 7**). Notably, Cluster 1 (including % of CD9-EVs, % of CD81-EVs, % of CD63-EVs, Lactate Dehydrogenase (LDH), ratio Neutrophil to Lymphocyte (NLR) and Stage IV) was significantly associated to ORR at univariate analysis. Furthermore, among variables in this cluster, high levels of CD63 positive EVs were correlated with the best ORR (Odds Ratio=1.63 per % unit increase; p=0.008 at multivariate, **Figure 5C**). Progression free survival of NSCLC patients was evaluated according to the cluster’s analysis. Multivariate analysis revealed that the presence of cluster 1 is associated with a small but significant reduction of disease progression compared to the other clusters (HR 0.58, per % unit increase p=0.0125, **Figure 5D**). The analysis of cluster with the overall survival revealed that the presence of cluster 1 was associated to a better prognosis in ICI-treated patients (HR= 0.62, p=0.0125, **Figure 5E**). These results highlight the potential of measuring tetraspanins levels on circulating EVs as a conceivable biomarker for response to ICI in NSCLC patients.

## Discussion

Research on EVs gained significant importance due to their diagnostic potential. Several evidences indicate that EVs derived from tumor cells are part of the cross-talk with nearby immune cells and could be used as biomarker for immunotherapies (36, 37). The use of immune-checkpoint inhibitors has radically changed the clinical practice in lung cancer but unfortunately only a subset of patients responds to therapy (38). Thus, the identification of biomarkers of response is a crucial clinical need. To date the immunohistochemical analysis of PD-L1 expression in tumor tissue and the tumor mutational burden are considered the best available biomarkers guiding ICI treatment. However, tissue biopsy is an invasive approach that could also lead to complications such as bleeding or infection and, most importantly, is not practicable while of no compliance for monitoring response to treatment (39). The finding of PD-L1 expresses in EVs from human urine or plasma and its correlation with disease progression in head and neck squamous cell carcinoma (40) and melanoma (27) prompted the investigation on whether it can similarly work in lung cancer. Although we confirmed that PD-L1 can be found on EVs, we were unable to correlate such expression with that on tumor tissue determined by IHC in NSCLC patients, as also observed in a previous study (30).

The surface profile of plasma EVs reflects the tissue-cellular sources secreting the vesicles and could be useful for the definition of their functional state. Generally, the different expression patterns of tetraspanins observed in EVs isolated from plasma of NSCLC patient’s reflect the relative amount of EVs in blood circulation (41). Interestingly, we observed significant differences between R- and NR- EVs considering the conventional EVs markers such as CD9, CD81 and CD63, and we found significant positive correlation of tetraspanins levels with ORR and PSF in patients. The modulation of CD9 may be associated to changes in platelet’s number or activation since this tetraspanin is highly present on EVs from these cells (42, 43). Also, differences in CD9 expression on EVs were observed in melanoma patients undergoing immunotherapy (44). Furthermore, the observed increase of CD81 expression in EVs from R patients of our cohort could be explained by the release of vesicles from B, T and NK cells (45) or by a different accumulation of these cells in R patients. This hypothesis is corroborated by the increased expression of NK-specific marker CD56 in EVs from R patients.

In our cohort, EVs isolated from the plasma of NSCLC patients expressed several immune markers and we found down-modulation of CD56 and HLA-ABC in EVs from NR patients highlighting a potential impairment of NK cells in these patients. Furthermore, anti-PD1 treatment likely modulated the cellular origin of plasma EVs in these patients. Indeed, the differences in CD56+ and HLA-ABC+ EVs observed at baseline between groups were completely abolished after ICI in favor of CD14+ vesicles, likely released by monocytes. The role of monocytes in the response to immunotherapy has found correlation in melanoma where the number of CD14^+^CD16^−^HLA-DR^high^ monocytes has been associated with better response to ICI therapy (46). We also found high levels of the epithelial marker EpCAM on the surface of EVs from R patients. Detection of soluble EpCAM in serum/plasma of cancer patients did not correlate with any clinical-pathological characteristics (47). Further investigations are needed to assess the role of EpCAM-EVs as diagnostic or prognostic biomarker in cancer.

However, the hypotheses on the different origin of EVs are based only on preliminary experiments and are at the moment only speculations. Further in-depth flow cytometry and molecular investigations should be performed to validate these findings in NSCLC patients before and during ICI.

Nevertheless, EVs and particularly exosomes, can exert opposite roles either stimulating or suppressing anti-tumor immune response in different conditions (48). Indeed, tumor-derived exosomes can be taken up by dendritic cells allowing them to internalize and cross present tumor antigens, for effective priming of tumor-specific T cells (48). Differently, tumor-derived exosomes possess the capacity to inhibit T cells directly (27, 29, 49) and to foster the function of myeloid-derived-suppressor cells (MDSC) (50) or suppressive neutrophils (51). Notably, exosomal PD-L1 can directly contribute to T cell suppression in both melanoma cancer patients and murine prostate and colon cancer models (27, 29). In our experimental setting, we showed inhibition of both CD4 and CD8 T cell proliferation, by lung cancer derived EVs from LT73. This result confirmed that tumor-derived EVs were able to modulate the phenotype and the activation of T cell as already demonstrated by other groups (52).On the contrary, EVs from NSCLC patients had no effect, or even increase T cell activation.

To our knowledge all the study describing the role of cancer EVs in immune modulation were performed using EVs isolated only from cancer cells (27, 29). Considering the whole EV’s population in the plasma of cancer patients, the relative amount of EVs secreted by cancer cells is very low. Based on these considerations, we are aware that EV enrichment and/or concentration is required to better elucidate the role of EVs from lung cancer patients in the modulation of immune cell activation. To solve this issue future studies will be performed by sorting specific EVs’ subpopulations and testing their functional activities. However, it is also possible that in NSCLC patients EVs do not have any functional property and that other molecular factors are the mediators of response to ICI. Nevertheless, despite their functionality, surface molecules expressed by EVs maintain their potential role of biomarkers for therapy.

Indeed, the results of our functional experiments between patient-derived EVs and T cells are in line with our findings showing that increased levels of EVs associate with improved clinical response, and can be explained by the expression of co-stimulatory molecules on EVs surface. Notably, despite increased in R-EVs, these molecules are also expressed in NR-EVs. This can account for their equal T cell promoting effect *in vitro*, where quantity of R-EVs and NR-EVs are comparable.

In conclusion, our results indicate that tetraspanins levels on EVs could represent promising biomarkers to select patients who will benefit from ICI regimen.

Our study had certain limitations. First, in Italy the recommendation for the treatment of NSCLC patients with TPS <50% is now the combination of chemotherapy plus immunotherapy so the efficacy of tetraspanin levels on EVs surface should be confirmed also in this therapeutic setting. Then, EVs isolation by ultracentrifugation and subsequently surface marker detection by flow cytometry is not feasible in a clinical setting. Furthermore, in this study we focused only on direct modulation of T cells by EVs, excluding their possible interplay with other immunosuppressive populations that will be the object of future investigations.

## Data availability statement

The raw data supporting the conclusions of this article will be made available by the authors, without undue reservation.

## Ethics statement

The studies involving human participants were reviewed and approved by Internal Review and the Ethics Boards of the Istituto Nazionale dei Tumori (INT 22-15 and INT 11-21) of Milan. The patients/participants provided their written informed consent to participate in this study.

## Author contributions

DS, PG, OF, CP and EJ carried out all the experiments and drafted the manuscript; FP, IP and VC performed the flow cytometry analysis, MB, AT, RF, GLR, AP, GV, MO, MG and GG collected blood samples and clinical data; AMF carried TEM; VT performed the statistical analysis; MCG, MC, and GS conceived of the study, and participated in its design and coordination and helped to draft the manuscript. All authors read and approved the final manuscript.

## Funding

The study was supported by grants from the Italian Ministry of Health (GR-2019-12369047 to O.F and GR-2016-02362484 to EJ) and Fondazione Cariplo (2018–0213 to EJ).

## Conflict of interest

The authors declare that the research was conducted in the absence of any commercial or financial relationships that could be construed as a potential conflict of interest.

## Publisher’s note

All claims expressed in this article are solely those of the authors and do not necessarily represent those of their affiliated organizations, or those of the publisher, the editors and the reviewers. Any product that may be evaluated in this article, or claim that may be made by its manufacturer, is not guaranteed or endorsed by the publisher.
