# Supplementary material for: Circulating CD81-expressing extracellular vesicles as biomarkers of response for immune-checkpoint inhibitors in advanced NSCLC
Source: Front Immunol. 2022 Sep 20;13:987639. doi: 10.3389/fimmu.2022.987639 (PMC9530186; doi:10.3389/fimmu.2022.987639)
Supplement: Supplementary file 1 [file Table_1.docx]

|  | **N** | **%** |
| --- | --- | --- |
| **Sex** | | |
| **Female** | 30 | 46.9% |
| **Male** | 34 | 53.1% |
| **Age** | | |
| **<65 years** | 28 | 43.8% |
| **>65 years** | 36 | 56.3% |
| **Performance Status** | | |
| **0** | 19 | 29.7% |
| **1** | 40 | 62.5% |
| **2** | 5 | 7.8% |
| **Smoking status** | | |
| **never** | 12 | 18.8% |
| **former** | 30 | 46.9% |
| **current** | 22 | 34.4% |
| **Stage** | | |
| **IIIb** | 2 | 3.1% |
| **IVa** | 40 | 62.5% |
| **IVb** | 22 | 34.4% |
| **Tumor Histology** | | |
| **ADC** | 48 | 75.0% |
| **SCC** | 11 | 17.2% |
| **Other** | 5 | 7.8% |
| **Status PD-L1 in tissue** | | |
| **<1%** | 38 | 59.4% |
| **1<49%** | 26 | 40.6% |

**Suppl. Table 1. Clinical characteristics of lung cancer patients**

|  | **N** | **%** |
| --- | --- | --- |
| **Immunotherapy** | | |
| **Atezolizumab** (anti-PD-L1) | 6 | 9.4% |
| **Avelumab** (anti-PD-L1) | 1 | 1.6% |
| **Durvalumab+Tremelimumab** (Anti PD-L1 + Anti CTLA-4) | 2 | 3.1% |
| **Nivolumab** (anti-PD1) | 24 | 37.5% |
| **Nivolumab+Ipilimumab** (anti-PD-1+anti-CTLA-4) | 1 | 1.6% |
| **Pembrolizumab** (anti-PD1) | 30 | 46.9% |
| **Line of immunotherapy** | | |
| **1** | 26 | 40.6% |
| **2** | 32 | 50.0% |
| **3** | 6 | 9.4% |

**Suppl. Table 2. Immunotherapy treatments of lung cancer patients**

**Suppl. Table 3. Clinical response of lung cancer patients**

|  | **N** | **%** |
| --- | --- | --- |
| **Hyper Progressor** | | |
| **no** | 48 | 80.0% |
| **yes** | 12 | 20.0% |
| **Best response** | | |
| **Progressive disease (PD)** | 35 | 58.3% |
| **Stable disease (SD)** | 12 | 20.0% |
| **Partial response (PR)** | 13 | 21.7% |
| **Prognosis** | | |
| **Alive** | 9 | 15.0% |
| **Dead** | 51 | 85.0% |

**Suppl. Table 4. List of antibodies used for flow cytometry**

| **Antibody** | **Clone** | **Fluorescence** | **Company** |
| --- | --- | --- | --- |
| Anti human CD9 | HI9a | FITC | Biolegend, cat. no. 312104 |
| Anti human CD81 | 5A6 | PE/Cyanine7 | Biolegend, cat. no. 349512 |
| Anti human CD63 | H5C6 | APC | Biolegend, cat. no. 353008 |
| Anti human CD274 (PD-L1) | MIH1 (RUO) | BV421 | BD Bioscience, cat. no. 563738 |
| Anti human CD4 | clone RPA-T4 | Alexa fluor 700 | ebioscience, cat. no 56-0049-41 |
| Anti human CD8 | clone RPA-T8 | Super Bright 780 | ebioscience, cat. no 78-0088-42 |
| Anti human CD279 (PD1) | clone eBioJ105 | Super Bright 600 | ebioscience cat. no 63-2799-41 |
| Anti human IFNg | clone 4S.B3 | APC-efl780 | ebioscience, cat. no 47-7319-41 |
| Anti human Granzyme B | clone GB11 | PE | ebioscience, cat. no 12-8899-41 |
| Anti human CTLA4 | clone BNI3 | BV786 | BD Biosciences, cat. no 563931 |
| Anti human CD86 | clone 2331 | PE-Cy7 | BD Biosciences, cat. no 561128 |
| Anti human ICOS | clone ISA-3 | APC | ebioscience, cat. no 17-9948-42 |
| Anti human CD137 (41BB | clone 4B4-1 | BV650 | BD Biosciences, cat. no 564092 |
| Anti human CD252 (OX40L) | clone 11C3.1 | PE | Biolegend, cat. no. 326308 |

**Suppl. Table 5. Experimental values**

|  | **N** | **Median** | **P25** | **P75** |
| --- | --- | --- | --- | --- |
| **% of PD-L1+ EVs** | 64 | 1.85 | 0.35 | 3.55 |
| **% of Epcam+ EVs** | 64 | 16.50 | 9.00 | 41.00 |
| **% of TIGIT+ EVs** | 64 | 14.70 | 9.17 | 28.94 |
| **% of PD-L2+ EVs** | 64 | 7.00 | 0.00 | 21.00 |
| **% of VISTA+ EVs** | 64 | 0.95 | 0.94 | 0.97 |
| **% of CD9+ EVs** | 61 | 9.71 | 5.87 | 17.50 |
| **% of CD81+ EVs** | 61 | 6.84 | 5.77 | 12.54 |
| **% of CD63+ EVs** | 53 | 0.64 | 0.51 | 1.21 |
| **Lactate dehydrogenase** | 56 | 353.00 | 299.50 | 415.50 |
| **Neutrophil/Lymphocyte ratio** | 62 | 4.39 | 2.78 | 6.80 |

**Suppl. Table 6. Univariable logistic analysis of association between selected variables and ORR**

| **Variable** | **Raw_p value** | **FDR_p value** |
| --- | --- | --- |
| **% of CD9+ EVs** | **0.00015** | **0.00270** |
| **% of CD81+ EVs** | **0.00051** | **0.00312** |
| **% of CD63+ EVs** | **0.00052** | **0.00312** |
| % of TIGIT+ EVs | 0.02601 | 0.11705 |
| NLR_ratio | 0.04915 | 0.17694 |
| % of PDL2+ EVs | 0.11416 | 0.34248 |
| PDL1_in tissues | 0.28153 | 0.50675 |
| % of VISTA+ EVs | 0.26706 | 0.50675 |
| BMI_Baseline | 0.22327 | 0.50675 |
| smoker | 0.27570 | 0.50675 |
| % of PDL1+ EVs | 0.31731 | 0.51923 |
| LDH_baseline | 0.45808 | 0.63426 |
| PS_1 | 0.44034 | 0.63426 |
| % of Epcam+ EVs | 0.57538 | 0.69046 |
| Sex | 0.57360 | 0.69046 |
| age>65 | 0.66731 | 0.75072 |
| ADC | 0.85771 | 0.90816 |
| Stage IV | 0.98099 | 0.98099 |

**Suppl. Table 7. Cluster procedure selection variables**

| **Cluster** | **Variable** | **OwnCluster** | **NextClosest** | **RSquareRatio** |
| --- | --- | --- | --- | --- |
| **Cluster 1** | % of CD9+ EVs | 0.8349 | 0.0029 | 0.1656 |
|  | % of CD81+ EVs | 0.9214 | 0.0305 | 0.0810 |
|  | % of CD63+ EVs | 0.8302 | 0.0159 | 0.1725 |
|  | LDH_baseline | 0.0274 | 0.1473 | 1.1406 |
|  | NLR_ratio | 0.1134 | 0.1507 | 1.0439 |
|  | Stage IV | 0.3799 | 0.0191 | 0.6322 |
| **Cluster 2** | % of Epcam+ EVs | 0.8104 | 0.0374 | 0.1970 |
|  | % of TIGIT+ EVs | 0.8128 | 0.0525 | 0.1976 |
|  | % of PDL2+ EVs | 0.3452 | 0.0158 | 0.6653 |
|  | % of VISTA+ EVs | 0.1015 | 0.0147 | 0.9119 |
|  | BMI_at baseline | 0.1374 | 0.0823 | 0.9399 |
|  | PS_1 | 0.1442 | 0.0226 | 0.8755 |
| **Cluster 3** | PDL1_in tissue | 0.4479 | 0.0107 | 0.5581 |
|  | % of PDL1+ EVs | 0.2367 | 0.0104 | 0.7713 |
|  | age>65 | 0.3183 | 0.0397 | 0.7099 |
|  | smoking | 0.7227 | 0.0301 | 0.2859 |
| **Cluster 4** | Sex | 0.6107 | 0.0032 | 0.3905 |
|  | ADC | 0.6107 | 0.0124 | 0.3942 |

**SUPPLEMENTARY FIGURE LEGENDS**

**Supplementary Figure 1. Overall Survival and Progression Free survival of NSCLC patients treated with ICI.** Kaplan-Meier curves reporting the progression free or overall survival of lung cancer patients stratified by the Response according to RECIST 1.1 criteria such as Response (R), Stable Disease (SD), Progression (P) or Hyperprogression (HP).

**Supplementary Figure 2.** **Phenotypic analysis of EVs at baseline. A)** Median fluorescence intensity of tetraspanin expression on circulating EVs from R and NR patients and HS controls, measured by flow cytometry as indicated in figure 1C. **B)** Median fluorescence intensity of tetraspanin expression on circulating EVs isolated from R and NR patients analyzed using Macsplex kit (R n=8; NR n=10) **C)** Profiles of surface EVs’ markers determined using Macsplex kit in R and NR patients. The values are the median fluorescence intensities (n=8 per group). **D)** Histogram comparing Tumor Proportion Score in Tissues and percentages of PD-L1+ EVs in patients.

**Supplementary Figure 3.** **Phenotypic analysis of EVs during therapy. A)** Mean Fluorescence intensity of CD9, CD63 and CD81 levels of EVs isolated by lung cancer patients and HS controls during ICI (HS=5 R BL n=8 vs; R TP1 n=6; NR baseline n=10 vs; NR TP1 n=6).**B)** Profiles of surface EVs’ markers determined by flow cytometry in R and NR patients at baseline (BL) and during therapy (TP1). The values are the median fluorescence intensities (R BL n=8 vs; R TP1 n=6; NR baseline n=10 vs; NR TP1 n=6).

**Supplementary Figure 4. Lung cancer cell lines-derived EVs impact on T cells functionality.** CFSE labeled CD8^+^ and CD4^+^T cells isolated from healthy donor PBMCs were primed *in vitro* with CD3/CD28, either alone (Ctrl +) or in presence of different concentrations (1, 5 or 15μg) of EV isolated from the LT73 cell line. Negative control T cells were left not stimulated (ns). Histograms report absolute numbers of CD8^+^ and CD4^+^ lymphocytes and proliferation (measured as CFSE dilution) and percentage of the indicated surface (PD1) or intracellular (IFNγ and GranzymeB) markers, within either CD8+ or CD4+ T cells.

**Supplementary Figure 5. Gating strategy used for flow cytometry analyses of T cells. A)** Gating strategy adopted for analyses of T cells reported in Figure 4/S4/S5. **B)** Representative flow cytometry histograms showing CFSE dilution in CD8^+^ or CD4^+^ T cells. Grey: non stimulated control, blue: activated cells. **C).** Representative flow cytometry histograms showing expression of IFNγ, PD1 and GranzymeB in CD8^+^ or CD4^+^ T cells. Black: Fluorescence minus one (FMO) control red: specific staining. **D)** Representative flow cytometry dot plot showing PD1^+^, IFNγ^+^, PD1^+^ and GranzymeB^+^ cells in CD8^+^ (left panels) or CD4^+^ (right panels) T cells. Stained samples and control FMO are shown.

**Supplementary Figure 6. R-EVs and NR-EVs equally affect T cells proliferation and activation.** Histograms referring to data shown in figure.4 report mean fluorescence intensity of the indicated surface (PD1) or intracellular (IFNγ and GranzymeB) markers, within either CD8+ or CD4+ T cells.
