# Supplementary material for: Circulating CD81-expressing extracellular vesicles as biomarkers of response for immune-checkpoint inhibitors in advanced NSCLC
Source: Front Immunol. 2022 Sep 20;13:987639. doi: 10.3389/fimmu.2022.987639 (PMC9530186; doi:10.3389/fimmu.2022.987639)
Supplement: Supplementary file 2 [file Presentation_1.pptx]

## Slide 1
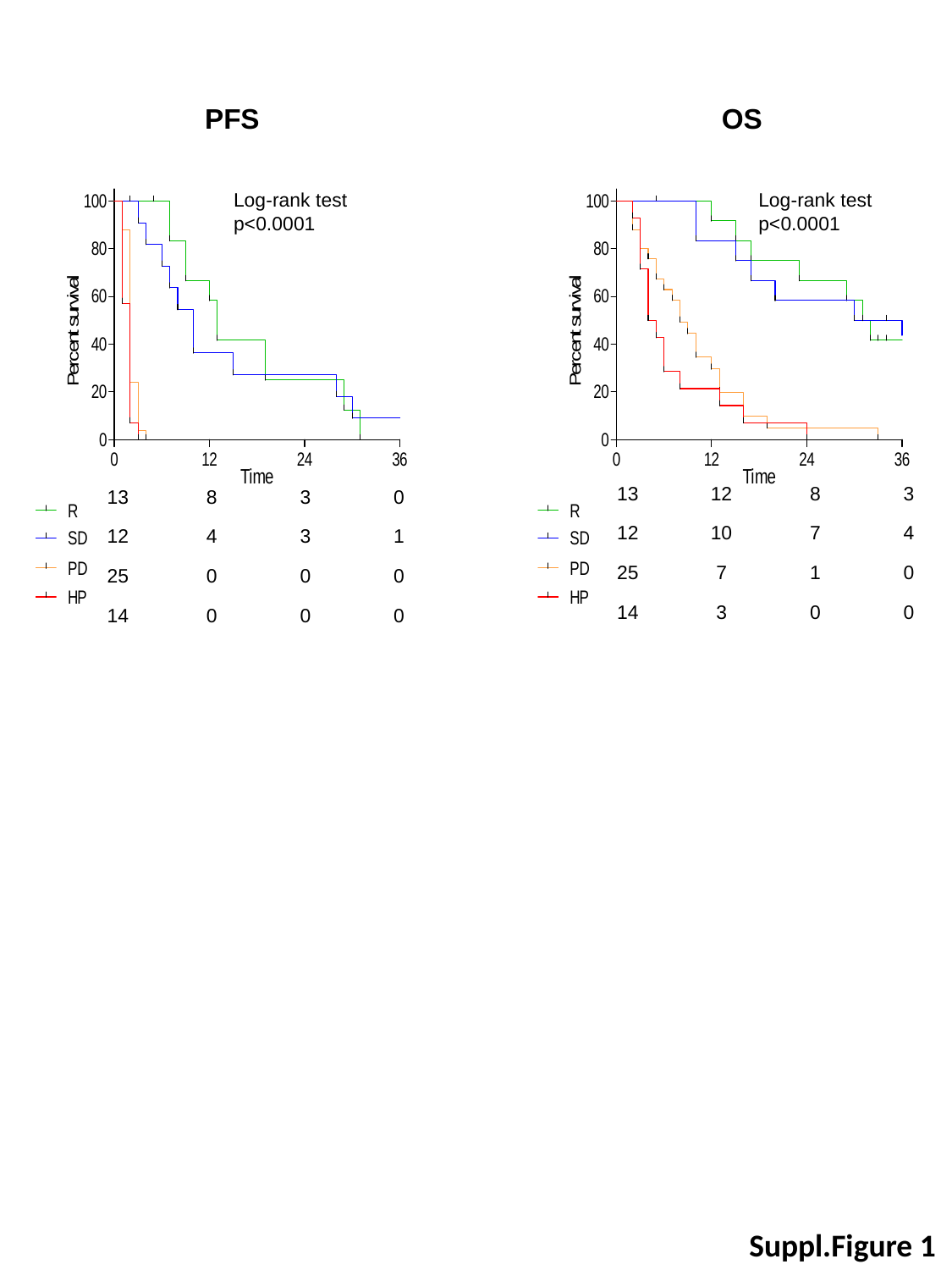

PFS
OS
Log-rank test p<0.0001
Log-rank test p<0.0001
| 13 | 12 | 8 | 3 |
| --- | --- | --- | --- |
| 12 | 10 | 7 | 4 |
| 25 | 7 | 1 | 0 |
| 14 | 3 | 0 | 0 |
| 13 | 8 | 3 | 0 |
| --- | --- | --- | --- |
| 12 | 4 | 3 | 1 |
| 25 | 0 | 0 | 0 |
| 14 | 0 | 0 | 0 |
Suppl.Figure 1

## Slide 2
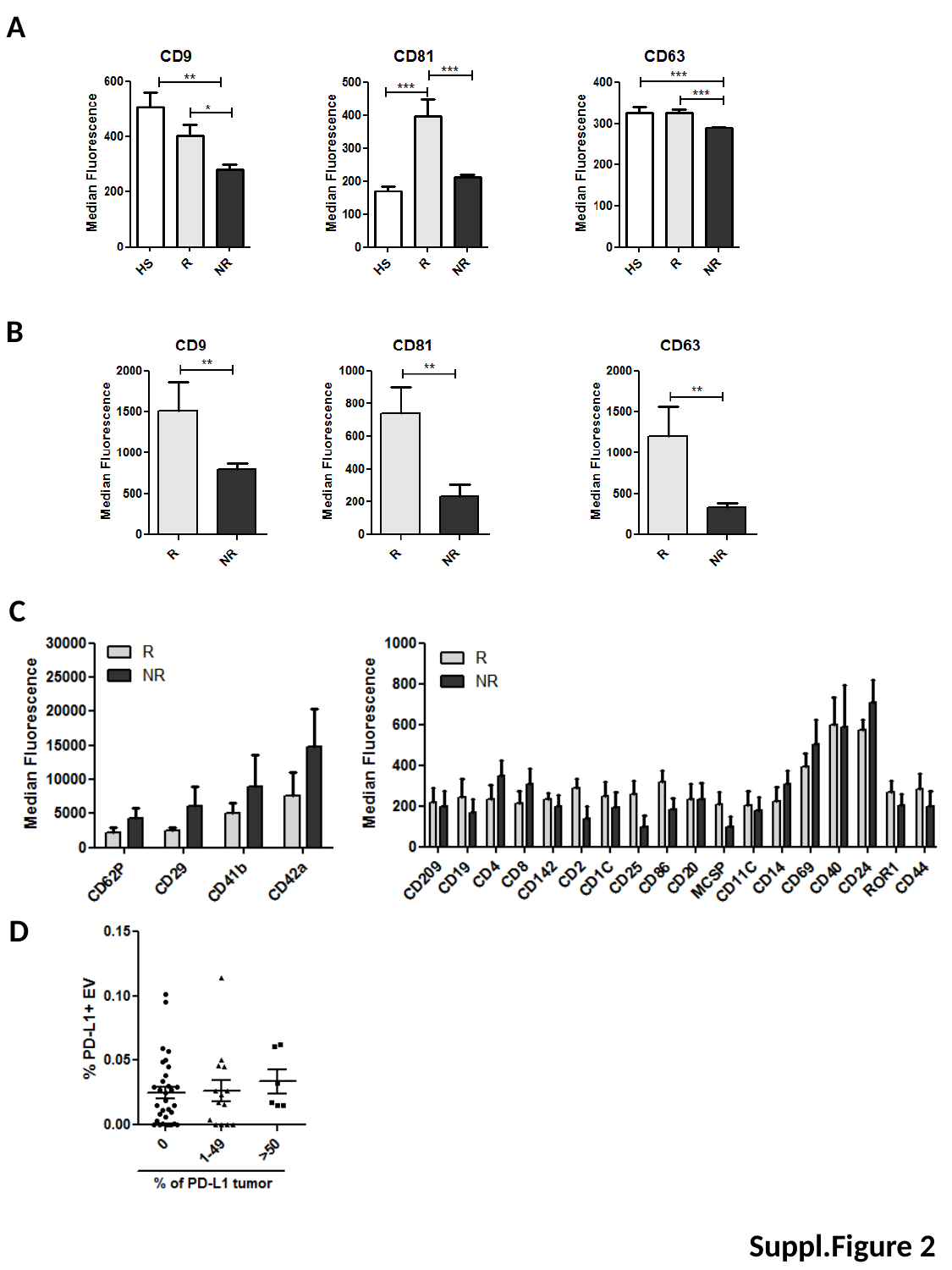

A
B
C
D
Suppl.Figure 2

## Slide 3
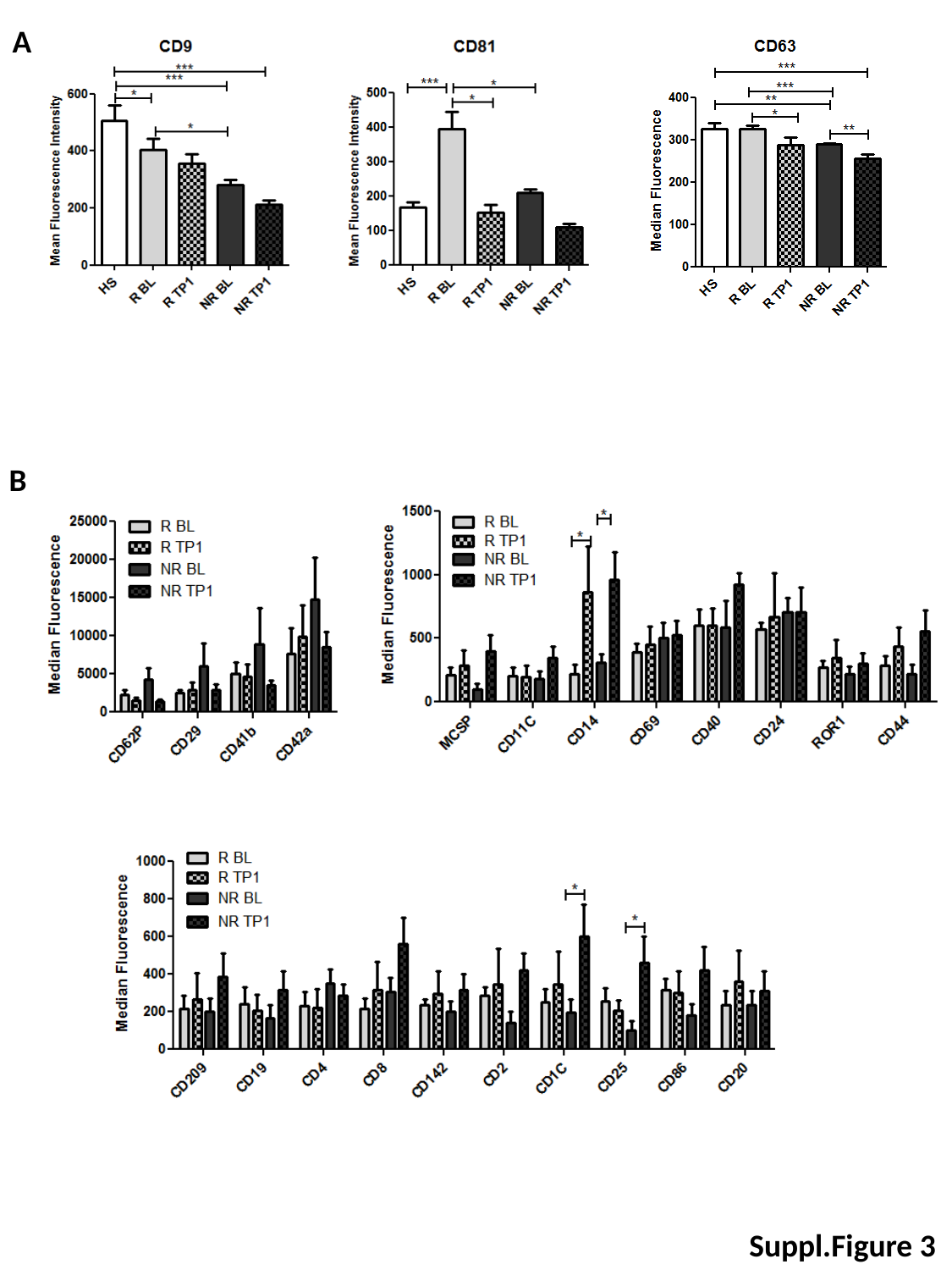

A
B
Suppl.Figure 3

## Slide 4
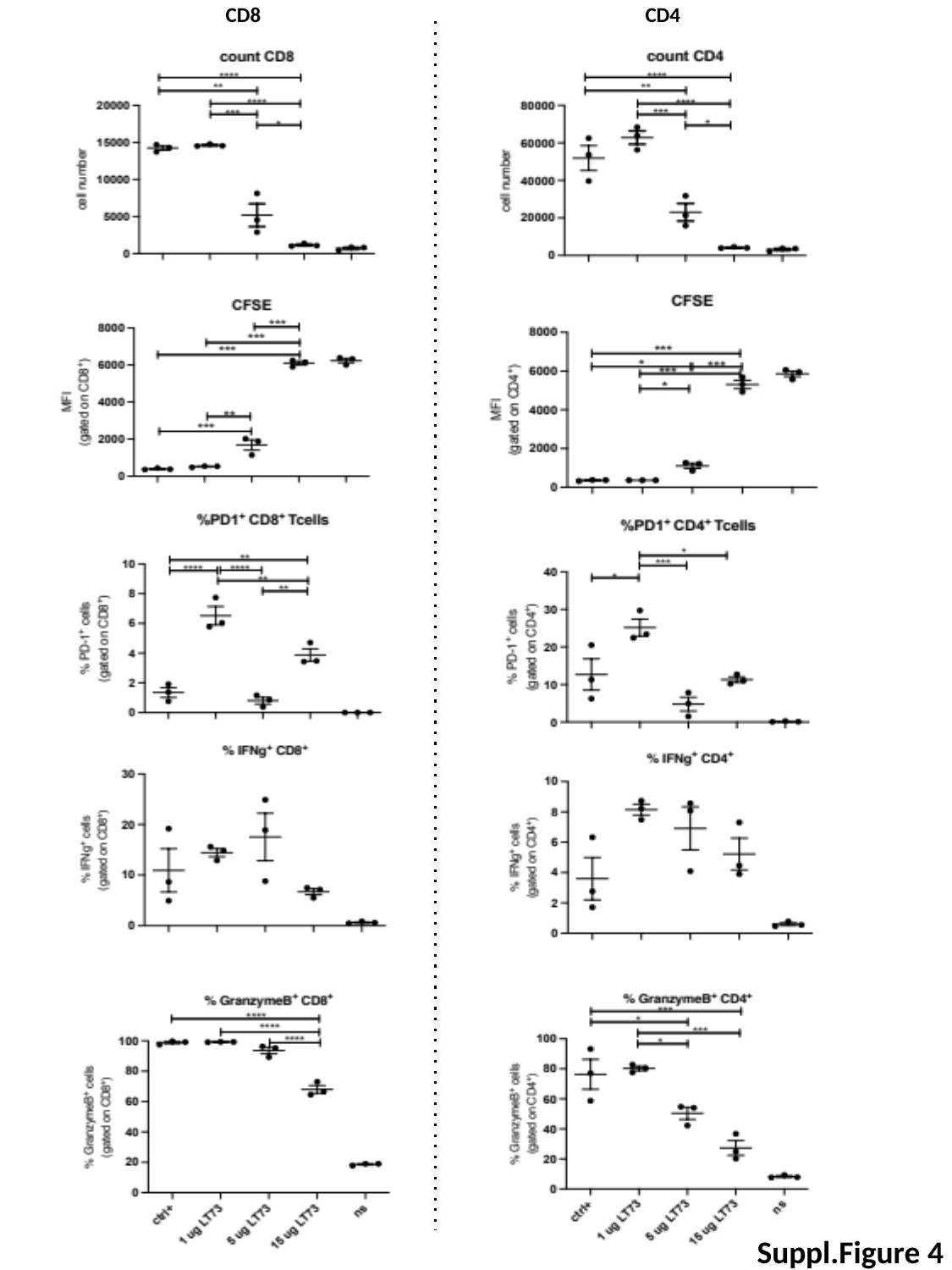

CD8
CD4
Suppl.Figure 4

## Slide 5
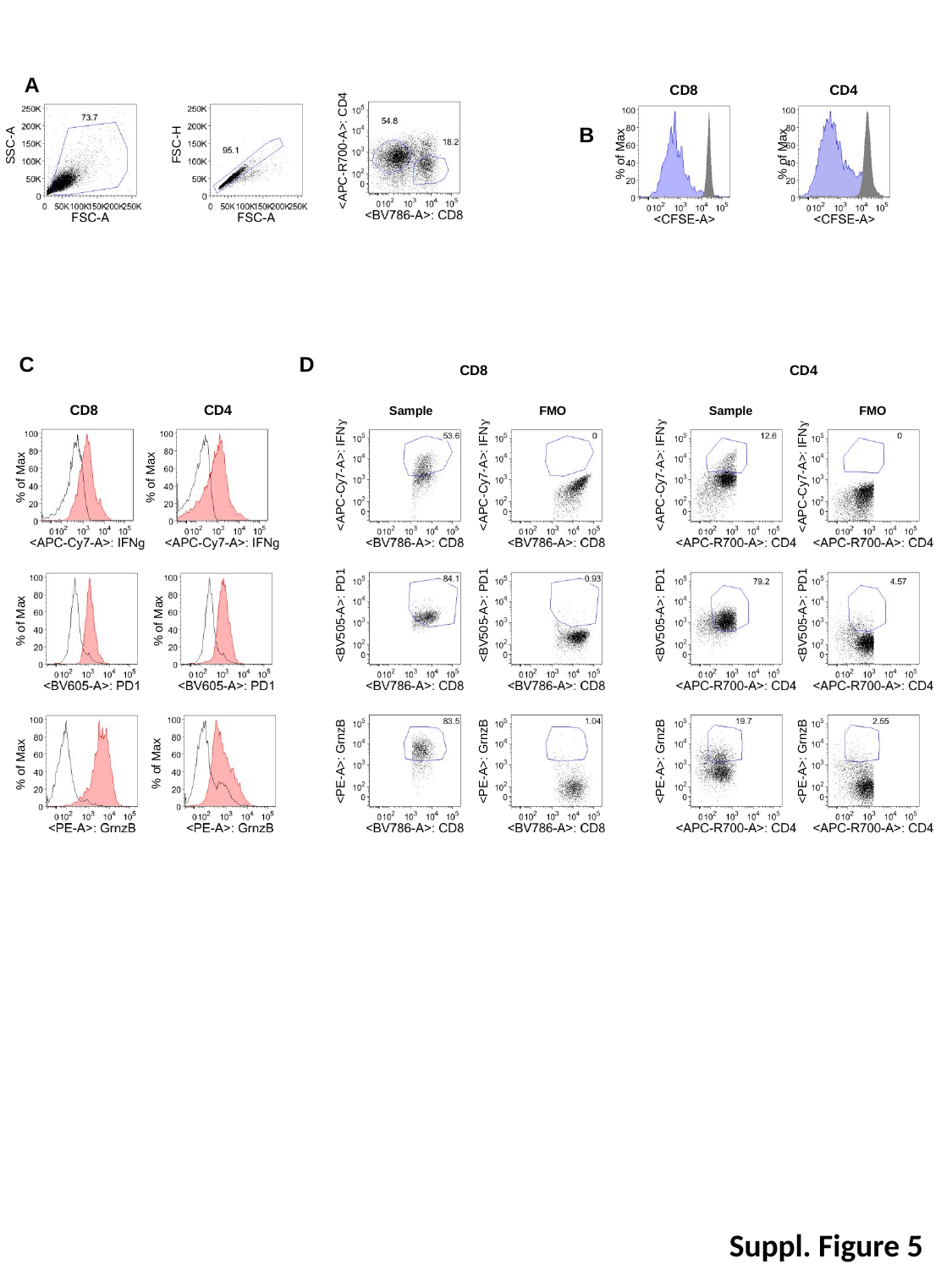

A
CD8
CD4
% of Max
% of Max
<APC-R700-A>: CD4
SSC-A
FSC-H
B
C
D
CD8
CD4
CD8
CD4
Sample
FMO
Sample
FMO
<APC-Cy7-A>: IFNg
<APC-Cy7-A>: IFNg
<APC-Cy7-A>: IFNg
<APC-Cy7-A>: IFNg
% of Max
% of Max
<BV505-A>: PD1
<BV505-A>: PD1
<BV505-A>: PD1
<BV505-A>: PD1
% of Max
% of Max
<PE-A>: GrnzB
<PE-A>: GrnzB
<PE-A>: GrnzB
<PE-A>: GrnzB
% of Max
% of Max
Suppl. Figure 5

## Slide 6
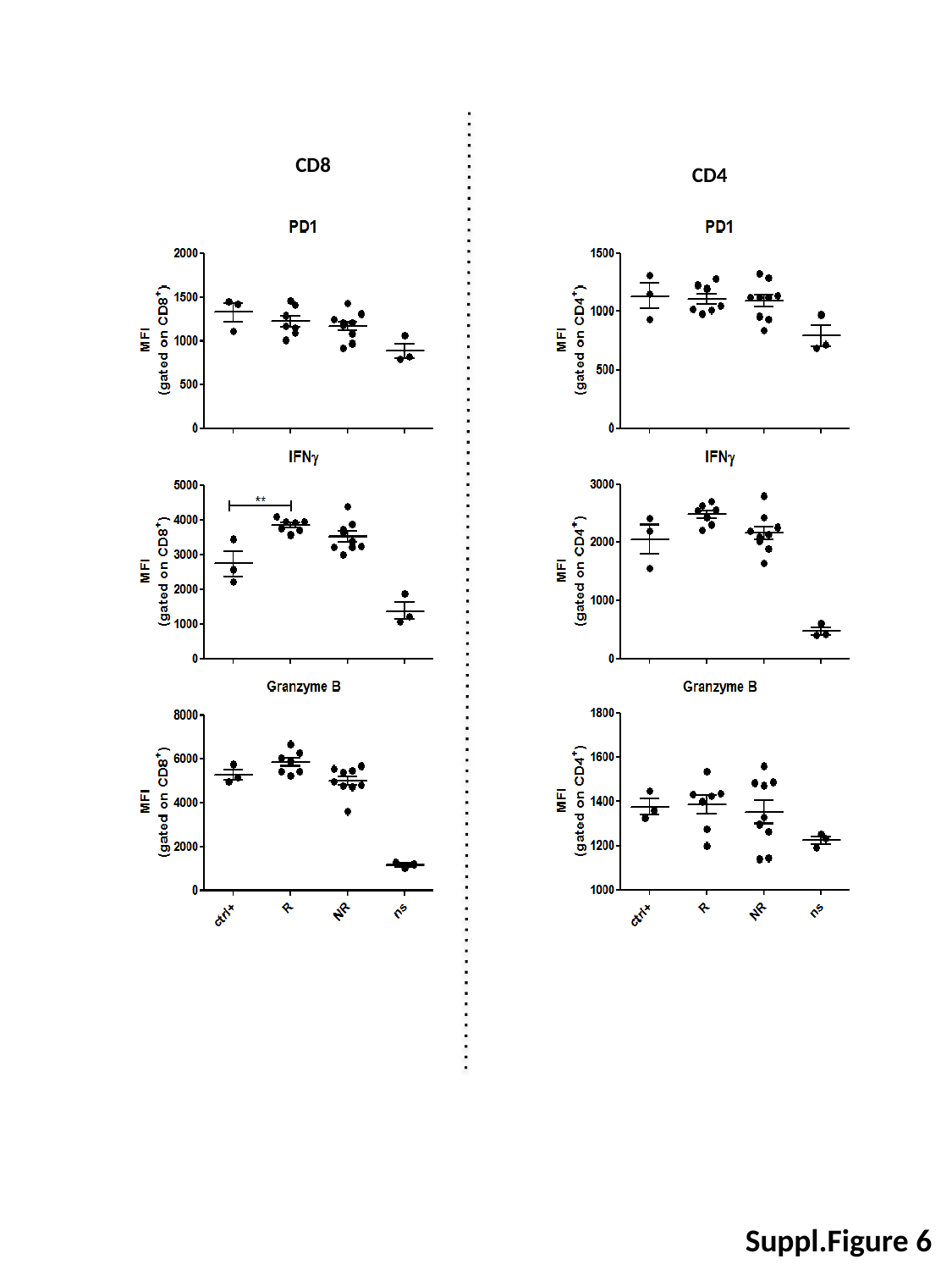

CD8
CD4
Suppl.Figure 6
